# Design of a Local Information Incentive Mechanism for Mobile Crowdsensing

**DOI:** 10.3390/s19112532

**Published:** 2019-06-03

**Authors:** Jose Mauricio Nava Auza, Jose Roberto Boisson de Marca, Glaucio Lima Siqueira

**Affiliations:** Center for Telecommunications Studies CETUC, Pontifical Catholic University of Rio de Janeiro PUC/Rio, Rio de Janeiro 22451-900, Brazil; jrbm@cetuc.puc-rio.br (J.R.B.d.M.); glaucio.siqueira@puc-rio.br (G.L.S.)

**Keywords:** mobile crowd-sensing, graph theory, game theory, incentive models, local information

## Abstract

The world of telecommunications has seen the growing popularity of mobile devices and its massive technological advancements and innovations (e.g., smartphones, smart watches, among others). One critical particularity is that these devices have a series of built-in sensors and continuous network connectivity. Therefore, they present a great opportunity to perform large-scale sensing of different activities in the physical world. This new sensor application, better known as Mobile crowd-sensing (MCS), has lately become a focus of research. One of the challenges when developing a MCS-based network is to attract and convince users to participate. In this paper, we present a framework for MCS that includes a model to represent the behavior of the users and a novel incentive mechanism. The model aims to characterize the behavior of users considering the availability of their resources and the non-homogeneity of their responses. The incentive mechanism proposed assigns different values of incentives and in it the users only consider their local information to decide their participation in the framework. The performance of the proposed framework is evaluated through simulations. The results allow us to prove the uncertainty of participation of the users and that they react in different ways to the incentives offered. They also prove that the incentive mechanism estimates satisfactorily the type of users and the incentive that will be offered to each user. In addition, we show the advantages of an incentive mechanism that considers different values of payments.

## 1. Introduction

Wireless sensor networks are one of the areas that have experienced a significative growth over the last few years. The advances in research and industry have brought more complexity to its applications, demanding the need for enhanced computing capabilities and a higher number of nodes to cover bigger areas. Note that a traditional sensor network for a large-scale sensing scenario needs a vast number of sensor nodes in order to possess the sufficient amount of data and guarantee the coverage. The installation and maintenance of these nodes in an urban or rural area could be quite complicated and will make the deployment of this solution more expensive.

At the same time, we witnessed the advances of mobile devices (e.g., wearable devices, smartphones, music players, tablets) and their increasing popularity. According to the information technology research and advisory company Gartner Incorporation the worldwide combined shipment of devices will reach 2.4 billion units in 2019 [1] proving the high demand of these devices.

One important characteristic of these devices is their ability to communicate. Almost all of them have internet access and can connect to other networks or devices. Another important characteristic of these devices is that they have many built-in sensors (e.g., compass, GPS, camera, microphone, gyroscope, accelerometer and proximity sensor, among others). With these sensors, it is possible to collect information of their users and their environments to generate collective knowledge of the physical world. This new sensing paradigm is better known as Mobile Crowd-Sensing (MCS). MCS can be classified into two main categories: Participatory sensing and opportunistic sensing. Participatory sensing needs the active participation of the users to obtain and share the required data (e.g., photographs, videos, measurement samples, monitoring of environmental variables, among others). Opportunistic sensing is more autonomous and does not need an active involvement of users (e.g., carrier information, Wi-Fi signal sensing, location sampling).

MCS offers several advantages and solutions to some constraints that the classic sensor networks have. For example, one of the biggest constraints in wireless sensor networks (WSN) is energy consumption. On the contrary, in MCS the users take care of charging their devices on a daily basis. Mobile devices have more storage and computing resources than a regular sensor. Networks can be deployed at a lower cost because the mobile devices are already present in all the potential areas the network will cover. MCS is more expandable because if the applications needs more nodes the network will just have to recruit more users.

MCS enables several applications [2] and recently has been the focus of several studies [3,4,5,6,7,8] Using resources of devices that are already in the field has opened a new range of possibilities. For example, monitoring the traffic congestion of a whole city [9,10,11,12], parking solutions [13,14,15], measuring pollution levels in an urban area [16,17] and healthcare solutions [18,19]. In the near future, the industry expects to add even more sensors to the mobile devices, which will bring a vast amount of applications that will enhance the management of cities and our daily activities. Although MCS is promising, there are issues to consider. There are privacy concerns since mobile devices belong to humans and sometimes the sensory data may be personal. In addition, when a mobile user takes part in MCS consumes his own resources (e.g., computer power, battery, data plan). Therefore, there is a need to encourage users to cooperate or take part in the MCS framework through a reward system. The user cooperation relies directly on the motivation related to intrinsic and extrinsic issues. The intrinsic motivation depends on the beliefs, feelings, likes, priorities or desires. The extrinsic motivation is defined by exterior reinforcements or punishments usually translated into cash or valuations. Therefore, to design an incentive model it is important to consider that each person will have different motivations and will react differently to the different incentives offered.

**Figure 1:**
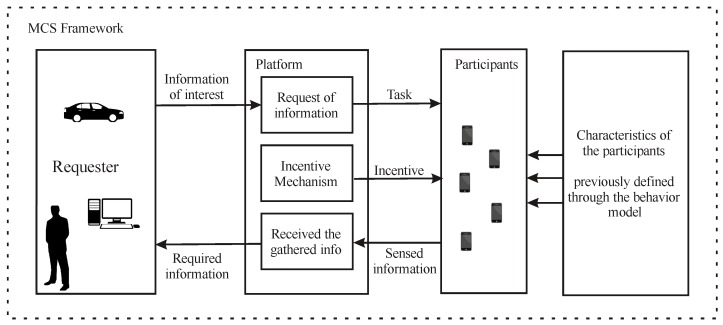
Proposed Framework for MCS.

In this work, we propose to model a technical framework for MCS that involves the modelling of the users’ behavior and an incentive mechanism for participatory MCS. The structure of our framework is illustrated in Figure 1. First, we propose a probability based method to define the intrinsic and extrinsic characteristics of each user. These characterizations allow us to model the participation decision of a user as a function of intrinsic and extrinsic motivations. Second, the platform starts sending the information requirements with the value of an incentive to recruit participants. The users that receive the requirement decide its participation based on their own motivations and how the incentive affects them.

An incentive mechanism is designed to determine a specific payment for each user. With the reaction of users to different payments, the platform will be able to determine which users are more sensitive to extrinsic incentives. Finally, the platform will choose a set of participants to achieve their goals.

More specifically, in the following points you can found the key contributions of this paper.

- We propose a model to represent abstractly the behavior of users. The model is based on probabilities to represent the uncertainty of users’ participation. With this model, we were able to represent and prove through simulations the non-homogeneous response of users to the different incentives.
- We present a novel game theory based incentive mechanism that assigns variable incentives considering the unique characteristics of each user. In it, users define its participation in the MCS framework considering only his own information. Different than many other incentive mechanisms of the literature where users need information of their neighbors to make a decision. It is important to point that the incentive mechanism has a process to achieve the participations goals of the platform.
- We evaluate the proposed MCS framework through simulations with different parameters to prove the performance of the proposed incentive mechanism.

This paper is organized as follows. Related works are introduced in Section 2. Section 3 gives the system model. We present the details of the problem formulation and the design of the incentive mechanism in Section 4. The simulations results are presented in Section 5. The summary and conclusions of the work are in Section 6.

## 2. Related Work

One of the first descriptions of MCS was made in [20] where the authors describe the growth of the mobile sensing and computing devices. They define MCS as a paradigm where individuals with sensing and computing devices can collectively share information to measure and map a phenomenon of common interest. MCS systems generally have a number of participants, the sensing application and the platform that will analyze the gathered information as presented in [21]. Given that MCS is an emerging topic, there are survey papers that seek to identify the characteristics and limitations of this paradigm [22,23,24,25]. Human involvement in these solutions comes with some limitations and opportunities that have been considered [26]. Many works of the literature identify the process of recruiting users as a critical issue since users are the core component of a MCS system. This challenging problem keeps attracting the interest of many researchers. According to several social and economic studies the human being is selfish by nature and what motivates him to take part of a collective action will be their personal interests [27]. These interests can be intrinsic (fulfill their own beliefs or desires) or extrinsic (monetary rewards or scores) [28,29]. The studies of incentives for MCS focus on the extrinsic incentives.

The literature studies two types of extrinsic incentives: Monetary and non-monetary. The growth of social networks, blogs and mobile phones are factors that popularize non-monetary incentives [30]. These technologies allow users to encourage each other. The rewards in this case will be the recognition of the community, new friends, reputation or personal satisfaction. However, when an activity brings no immediate benefit to the user, monetary rewards can be a powerful influence. In [31] the authors design an incentive model based on game theory. In which they define a scenario of *n* participants. These participants share their own information and the information of their neighbors. Authors model this process as a normal game where the usefulness of cooperating participants depends of two variables, one representing effective incentive and the other a punishment, and the sum of both determines the ultimate utility. Other game theory approach used to design incentive models is the Stackelberg game [32]. The game has two phases: The leader decides first, and the other player makes their decision considering the original decision of the leader. They use the game because of its similarity with the process of assigning a task to the participants [33]. In [34] the authors design an incentive mechanism through a reverse auction; participants interested in taking part submit a price proposal to perform this task.

Although all of these works present interest solutions to the problem of recruiting users to take part in MCS, none of them considers that the response of users to the incentives is not homogeneous. Also, most of the solution consider that the participants use information of other participants for taking their decisions. Gather information of other users is not always possible and results in a more complex deployment. To address these issues, we aim to project an incentive mechanism where users only consider his local information to define its actions and that they do no react homogenously to the different incentives.

## 3. System Model

**Figure 2:**
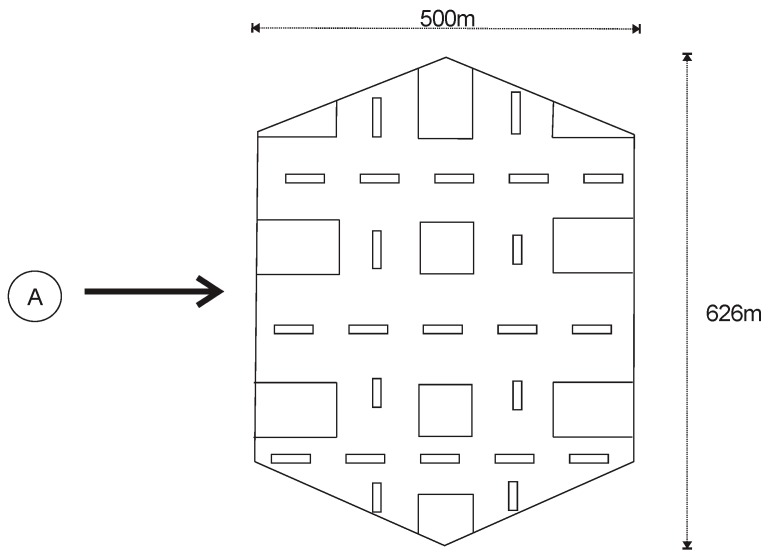
Physical location of a city represented as a vertex.

**Figure 3:**
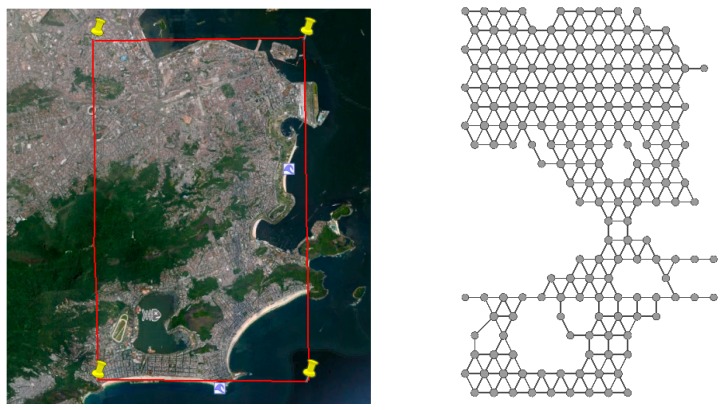
Area of interest in the city of Rio de Janeiro and its representation as a graph.

Consider an area of interest that will be represented as a graph where the vertexes will be the physical locations that the users or participants can visit. To represent a physical location as a vertex we consider that every city is full of mobile radio base stations and that their coverage is theoretically represented as a hexagon. Considering this principle, we cover the area of interest with hexagons. Therefore, in an area of interest we will have an amount of *A* physical locations. Figure 2 shows an example of a physical location and illustrates the size that each vertex will represent. Figure 3 shows the area of interest in a map and the representation of the same as a graph.

Consider a platform that needs information on several events *E*. An event will be a phenomenon of interest (e.g., measurement of pollution levels, monitoring of environmental variables, photographs of roads, among others). Each event is indexed by j∈{1, 2,…E}, and is directly related to a physical location.

A set of *N* potential collaborators will collect the information, consequently creating a collective sensing network. Each user *i* of this set i∈{1, 2,…N} has different abilities and interests. What differentiates users is the sensitivity to the reception of extrinsic incentives and their beliefs, feelings, priorities or desires that determine their internal or intrinsic motivation.

At certain instants of time, the platform requires information of an event of interest *E* in different physical locations, a∈{1, 2,…A}, of the analyzed area and offers an incentive or payment $K_{i}$ to motivate user participation. Participants receive the request and decide if they will perform the sensing. If they participate, they send the requested data to the platform and later receive their incentive.

In this case of study the platform will not define a budget constraint to encourage the contribution of the different users *i* in the different events *j*. One hypotheses of this work is that the participation of users in a task varies according to the degree or amount of extrinsic incentives presented to it. Considering this, each user responds differently to diverse incentives. In addition, it is important to consider that some users are more susceptible than others are to smaller payments, while others only react to more significative offers.

## 4. Formulation Problem and Incentive Model Design

In this section, we first model the behavior of users in detail. Then we analyze the problem and show how we can represent it in a game theory perspective. Finally, we design our incentive model. For easy reference, in Table 1 we list the important notation used in this section.

### 4.1. Modelling Users Behavior

In different approaches observed in the existing literature, the authors consider the non-participation of a user as a strategic issue of the same. They do not consider that for any user this application or approach may or may not be a priority and that the availability of resources (time, data packet, battery) to take part in collective sensing is variable. The lack of participation should not always be considered as a lack of interest. To model the decision of participation of a user we propose a probabilistic model with the following concepts:

**Definition** **1.** *We will define the participation of a user i, in a crowd-sensing network as a probability that considers the personal motivations (intrinsic and/or extrinsic) of each user. The sum of both motivations will give the cooperation probability of a user i:*

(1) $$P_{cooperation}{}_{i}=f\left(Motivation_{intrinsec}{}_{i}\;+\;Motivation_{extrinsic}{}_{i}\right)$$

**Definition** **2.** *The amount or quantity of intrinsic and extrinsic motivations are modeled as probabilities and are given by:*

(2) $$P_{MI}{}_{i}=\gamma_{i}\times P_{I}{}_{i}$$

(3) $$P_{ME}{}_{i}=\;\phi_{i}\times P_{E}{}_{i}$$

*where*$P_{I}{}_{i}\;e\;P_{E}{}_{i}\in \left[0,\;1\right]$*are the probability of occurrence of the cooperation since the motivation is intrinsic or extrinsic, respectively. The variables*$\gamma_{i}$*and*$\phi_{i}$*represent the weight that each person gives to its own motivations and they satisfy the following condition:*$\gamma_{i}+\;\phi_{i}=\;1$.

**Definition** **3.** *The participation of users in a task varies according to the degree or amount of extrinsic incentives presented to them. The variable* K∈[0, 1] *represents the existence of the incentive and affects directly the extrinsic motivation:*

(4) $$P_{ME}{}_{i}\to K_{i}\times P_{ME}{}_{i}$$

Consequently, from Definitions 1–3 we have:

(5) $$P_{{cooperation}_{i}}=\gamma_{i}\times P_{I}{}_{i}+K_{i}\times \left(\phi_{i}\times P_{E}{}_{i}\right)$$

Each participant or user *i* will have a previously defined extrinsic motivation ($\phi \times P_{E}$) and intrinsic motivation ($\gamma \times P_{I}$), this way our model will consider a population that adopts different behaviors to the different incentives offered. The probabilistic approach allows us to simulate the resources availability of a user, thus achieving an abstract representation of the real world.

### 4.2. Formulation Problem

The main idea of this incentive model comes from the following analysis: it is possible to represent the system model by a Game Theory [35] perspective. The game will consider two players, the platform and the participant user. The set of actions or strategies will be cooperation or non-cooperation for the user and request information or not request it for the platform. The payment offered by the platform to carry out the collection of information will define the utility of the participant.

**Figure 4:**
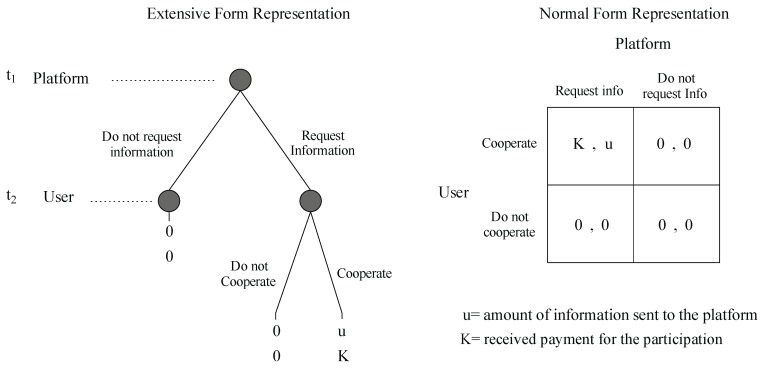
Game theoretic representation of our problem in normal and extensive form.

The left side of Figure 4 shows the game in its extensive form. Here, the game is represented as a tree, which nodes represent a decision point and the edges the actions. The variables at the end of a terminal node represent the payoffs. The right side of Figure 4 shows the game in its normal form matrix representation. We can see the two players one in the row, the other in the column and the actions they can play. In each cell, the first variable represents the payoff to the row player, and the second variable represents the payoff to the column player.

We can observe that the utility of the platform will be the amount of information received and the utility of the user the payment offered by the platform. We will analyze this game with two familiar concepts of game theory: The Nash equilibrium theorem and the concept of dominant strategies. A dominant strategy for a player will be the one that grants the highest payoff among all possible strategies for every possible action taken by other players. While, a pure Nash strategy is a set of strategies in which the strategy of each player is the best response in relation to the strategies of other players. Analyzing this game with these concepts, we can see that the only solution for both users will be to play cooperation for the user and request information for the platform. If this is the case, any rational participant user will conclude that the best option will be to participate in every opportunity he has, regardless of the value of incentive *K*.

**Figure 5:**
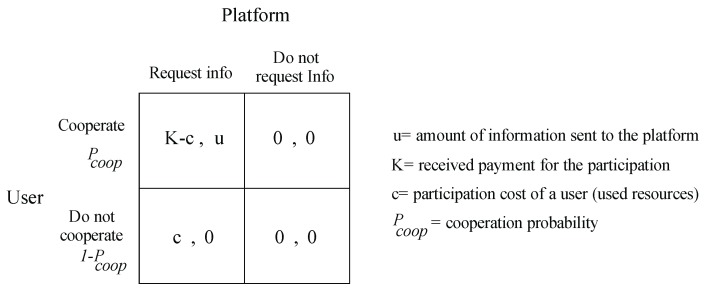
Game theoretic representation of our problem as a mixed strategy game.

We consider that the participation of a user has an abstract cost (time, battery, availability), which will be represented by the variable *c*. For a more realistic approach, we consider that the user only agrees to participate when the incentive offered *K* is greater than the cost of participation *c*, which depends directly on the activities of the person, the use of the device and personal motivations. This makes the variable *c* personal and different for each user. Figure 5 shows the updated game model where we see that our case of analysis is now a game with mixed strategies in which the player instead of choosing a profile of pure strategies, it will choose a distribution of probabilities on the same.

This probability distribution can be seen as the decision of participation for a user and we will represent it through the probability of cooperation presented in Equation (5).

Another characteristic of our approach is that the participants of the game follow a sequence. First, the platform requests the information and forwards the payment or incentive offered, immediately after, the user knowing the request of the platform and the offered payment can decide on the participation or not considering this information. On a future occasion, the platform may need to collect information from the same user, this can happen in more than one occasion. A game that is repeated over time is better known as a repetitive game.

We consider that the platform will need more than one request and that more than one user will attend this request. Therefore, the total cost of the system for the platform will be equal to:

(6) $$K_{Total}=\overset{E}{\underset{j=1}{\sum }}\overset{H}{\underset{l=1}{\sum }}K_{lj\;}$$

Here, *E* represents the number of requests of the platform and *H* the number of users that take part of the system sharing the requested information.

To define the utility of the game (Figure 5) for the user and the platform it is necessary to consider the definition of a mixed strategy game. The main idea of a mixed strategy game is that each player or user has a probability distribution over the set of actions. Let us consider the definition of a strategy $s_{i}$ for a user *i* as any probability distribution on the possible actions $A_{i}$. The total possible strategies for a user *i* are defined as $S_{i}$ and the set of all strategy profiles of a game is defined as the Cartesian product of the possible strategies of each user, $S=S_{1}\times S_{2}\times \cdots \times S_{n}$. Finally, it is necessary to know how to define the utility in these games because it is not possible to get the simple reading of the payments matrix since the game will not always end up in the same cell. To obtain the utility of a user $u_{i}\left(s\right)$ that has a mixed strategy profile s∈S, it is necessary to first calculate the probability of reaching each outcome given the strategy profile, and then calculate the average of the payoffs of the outcomes weighted by the probabilities of each outcome. Formally, we define the utility in a mixed strategy game as follows:

(7) $$u_{i}\left(s\right)=\underset{a\in A}{\sum }u_{i}\left(a\right)P\left(a|s\right)$$

where:

(8) $$P\left(a|s\right)=\underset{j\in N}{\prod }s_{j}\left(a_{j}\right)$$

Applying the Equations (6) and (7) to our game in Figure 5 we will have that the theoretical utility of a user will be equal to:

(9) $$u_{i}\left(s\right)=P_{cooperation}\times P_{inf}\times \left(K-c\right)+\left(1-P_{cooperation}\right)\times P_{inf}\times c$$

For this case, we will consider that $P_{inf}=1$, because the game will never exist if the platform decides to not request information to a user. If we represent the Equation considering that each user will have a finite quantity of participation attempts $Z_{i}$, we will have:

(10) $$u_{i}\left(s\right)=P_{cooperation}\times \overset{Z_{i}}{\underset{j=1}{\sum }}(K_{j}-c_{j})+\left(1-P_{cooperation}\right)\times \overset{Z_{i}}{\underset{j=1}{\sum }}c_{j}$$

To reach the definition of the utility of the user for our case of study we will make the final considerations. The Equation (9) is only fulfilled when utility values (*K* and *c*) are fixed over time. As previously defined, the variable *c* represents the cost of participation of the user, characteristic that is difficult to measure. That is why we will disregard the same and focus only on the participation payment. Therefore, the upper bound of the summation will be the number of successful participations *F*. The purpose of the variable *c* is to demonstrate that the user responses are not homogeneous over time, characteristic the model still represents through the probability approach of the user’s decision of participation presented in Equation (5).

Finally, we will disregard $P_{cooperation}$ because this information is unknown for the platform and for the model. Therefore, the total utility of the user will be as follows:

(11) $$U_{i}=\overset{F}{\underset{j=1}{\sum }}K_{j}$$

Likewise, we can define the total utility of the platform, but in this case, the platform has to consider the total quantity of responses that obtained in each event:

(12) $$U_{p}=\overset{E}{\underset{j=1}{\sum }}\overset{H}{\underset{l=1}{\sum }}u_{lj}$$

**Figure 6:**
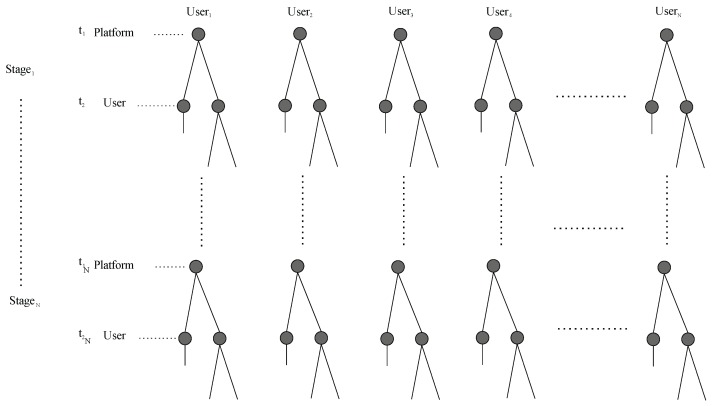
Extensive form representation of the amount of games that the platform will have with the different users.

In summary, our scenario will comprise an *N* number of users and each of them will play an independent game with the platform. In Figure 6, we can observe that the platform will play a game *E* times with *H* users. It is important to clarify that the number of participant users *H* may be different for each event *l*.

This process of strategic interaction allows us to create a history of the behavior of players. If the game does not update the incentive over time, the result of the same will be identical for all the repetitions. In this case, the platform will perform an update of the offered incentive *K* for each replay of the game. The incentive *K* will be variable mainly because each user reacts in different ways to the incentives offered and thus, the platform can save its resources avoiding maximum payment for all users. *K* affects directly the motivation of each person and consequently the probability of cooperation in Equation (5). *K* will be updated until the behavior of the user meets the platform requirements. Once the user reaches the requirements, platform will fix the amount of the payment *K* and the behavior of the user will stay stable. As the platform is the one that starts the game and requests the information, it will have control of the game and decide the amount of opportunities that a user takes part in the game.

### 4.3. Incentive Mechanism Design

The platform does not know how users react to different incentives. The only information that the platform has and can store are the responses of users to the different offered payments. Analyzing this data, the platform will define the value of the payment *K* that will grant to each participant for achieving the system requirements.

The value of the offered payment *K* for each user *i* in each participation will depend only on the behavior of user *i* over time. The variable σ will modify the offered payment and is calculated by the following expression:

(13) $$\sigma_{i}=sta_{i}\times \left[\left(inc\times \left(1-r_{i}\right)\times l_{i}\times m_{i}\right)-\left(dim\times r_{i}\times l_{i}\times d_{i}\right)\right]$$

where:

$sta_{i}=$ binary variable that represents if the payment *K* has been fixed or continues variable for the user *i*
inc= maximum value of increment for the payment *K*
dim= maximum value of diminution for the payment *K*
$r_{i}=$ cooperation rate of user *i*
$l_{i}=$ binary variable that considers if the minimum number of interactions between the platform and the user *i* has been met
$d_{i}=$ binary variable that indicates when the payment for the user *i* should be decreased
$m_{i}=$ binary variable that indicates when the payment for the user *i* should be increased

In the paragraphs below are presented the definitions of all the introduced variables in Equation (13).

The variable r in Equation (13) represents the response of user *i* to the different offered payments and we consider it as the cooperation rate or as the reputation of the user. There are reputation metrics created from a probability density function (PDF). When the analyzed events are binary in nature, the Beta PDF can be used to compute them [36,37]. The parameters of a PDF Beta can represent the behavior of the users.

The Beta distribution f(p|α,β) can be expressed through the gamma function:

(14) $$f\left(p|\alpha ,\beta \right)=\frac{\Gamma \left(\alpha +\beta \right)}{\Gamma \left(\alpha \right)\Gamma \left(\beta \right)}p^{\alpha +1}{\left(1-p\right)}^{\beta -1},\;\mathrm{where}\;0\leq p\leq 1,\alpha >0,\beta >0$$

where α represents the number of positive interactions (participation or cooperation), β the number of negative interactions (no participation) and p the probability of cooperation of the user.

The expected value of the Beta distribution is given by:

(15) $$E\left(p\right)=\frac{\alpha }{\alpha +\beta }$$

Therefore, the probability density function for analyzing the participation of users in a future can be expressed through an analysis of past observations. Considering that the parameters *a* and *b* count the positive and negative interactions, respectively.

(16) α=a+1    e     β=b+1 , where a,b≥0

For example, if we consider a user that sends the information seven out of ten times, we will have α=7, β=3 and E(p)=0.7. We can interpret the expected value as follows, although the relative frequency of a user participation is uncertain, the most likely value is 0.7.

Therefore, our cooperation rate will be equal to:

(17) $$r_{i}=E\left(p\right)=\frac{\alpha_{i}}{\alpha_{i}+\beta_{i}}$$

The platform defines a reference range of cooperation rate for each user *i,* $\left[lim_{inf}{}_{i},\;lim_{sup}{}_{i}\right]$, and if the cooperation rate $r_{i}$ is within this range, the platform considers the behavior of user *i* acceptable. Otherwise, when the cooperation rate $r_{i}$ is above the upper reference limit,$\;lim_{sup}{}_{i}$, or below the lower reference limit,$\;lim_{inf}{}_{i}$, the amount of the offered payment will be updated for the next attempt of participation. Equation (13) seeks the value to modify the payment *K* to achieve the satisfactory participation rate.

The constant variables *inc* and *dim* are the ones that define the value of σ. The same that can be positive or negative. If positive represents that the platform will increase the offered payment *K* otherwise *K* will decrease. The value of *dim* is calculated through the following relationship:

(18) $$dim=\{\begin{array}{ll} inc\times \frac{1-r_{desired}}{r_{desired}},\; & r_{desired}\geq 0.5\;\\ inc,\; & r_{desired}<0.5 \end{array}$$

As previously defined *inc* is a constant that defines a value to increase the offered payment *K* and $r_{desired}$ is a constant previously defined that represents the percentage of participation that the platform wants to achieve. In other words the desired rate of participation of the system. The direct relationship between these variables and how they affect Equation (13) defines the actions that the platform takes in relation to the value of the offered payment. Let us see the interaction between these variables with some examples:

Consider inc=0.25, $r_{desired}=0.5$ and r=0.5 we will have:

$$dim=inc\times \frac{1-r_{desired}}{r_{desired}}=0.25\times \frac{1-0.5}{0.5}=0.25$$

Considering a simplified version of the Equation (13), we will have:

σ=(inc×(1−r)−dim×r)=(0.25×(1−0.5)−0.25×0.5)=0

Analyzing this scenario, we can see that our model works properly because there would be no increase in the offered payment given that with the previously payment our participation rate reaches the desired participation rate of the platform.

Let us analyze what happens if r=0.7:

σ=(inc×(1−r)−dim×r)=(0.25×(1−0.7)−0.25×0.7)=−0.1

And if r=0.35:

σ=(inc×(1−r)−dim×r)=(0.25×(1−0.35)−0.25×0.35)=0.0750

With these examples, is possible to observe which guideline the platform will use to update the offered payment *K*. If the cooperation rate of a user *i* is greater than the desired rate, σ is negative and it decreases the offered payment *K* and if the opposite happens (cooperation rate of user *i* is lower than the desired rate), σ is positive and it increases the offered payment K. Also, the lower the value of r in relation to the desired rate, the greater the value of the increment. Likewise, the greater the value of r in relation to the desired rate, the greater the value of the subtraction. This way we prove that the update of the offered payment depends only of the behavior of the user *i* and the platform requirements.

When the update of the offered payment *K* occurs, it is fixed by a minimum amount of participation attempts *M*, and will only have a new update when the cooperation rate r is out of the range of the desired rates and the amount of participation attempts cnt with the specific payment *K* is equal or greater than *M*. The value of the constant *M* will determine the speed at which the platform performs updates of offered payment *K* and consequently the time it takes the platform to find the payment that makes the user achieve the desired cooperation rate constraints. In Equation (13), we model this behavior through the binary variable *l*:

(19) $$l=\{\begin{array}{c} 1,\;\;cnt\geq M\\ 0,\;\;cnt<M \end{array}$$

We need a new comparison criterion to define when the platform will perform a new payment update. Since if we just use the comparison of $r_{i}$ with the defined range of accepted rates like in the first rounds of interactions, it will not be possible to conduct a conclusive analysis since the value of the rate r represents the totality of participations of user *i* and does not reflect how user *i* reacts to a specific payment. What determines a new update will be the behavior of the user to the fixed payment. Therefore:

(20) $$r_{decision}=\frac{\alpha_{K_{i}}}{M}$$

Here, $\alpha_{K_{i}}$ is the amount of participations of user *i* with the payment *K*. As previously defined *M*, is the minimum amount of participation attempts for user *i* with the payment *K*. The binary variables *m* and *d* allows us to model this constraint in Equation (13).

(21) $$m=\{\begin{array}{c} 1,\;r_{decision}<lim_{inf}\\ 0,\;r_{decision}\geq lim_{inf} \end{array}$$

(22) $$d=\{\begin{array}{c} 1,\;r_{decision}\geq lim_{sup}\\ 0,\;r_{decision}<lim_{sup} \end{array}$$

The variable *m* acts as a flag in the Equation (13). Specifies when the platform can increment the payment while *d*, specifies when can decrease the payment.

For our model, one of the main objectives of the platform is to find a specific payment for each user. To avoid a very large number of payment updates, we need stability criteria. When a user reaches these criteria, the variable *sta* is equal to zero and as presented in Equation (13), it will avoid future updates. In our case, the stability criteria will be the following:

- When the cooperation rate *r* or the decision cooperation rate $r_{decision}$ are within the established bounds ($lim_{inf}$ and $lim_{sup}$)
- When a payment decreases, results in an $r_{decision}$ below $lim_{inf}$. In the next attempt, the platform will increase the offered payment for the last time and, no matter what happens in the future it will continue with the same payment *K*.

**Figure 7:**
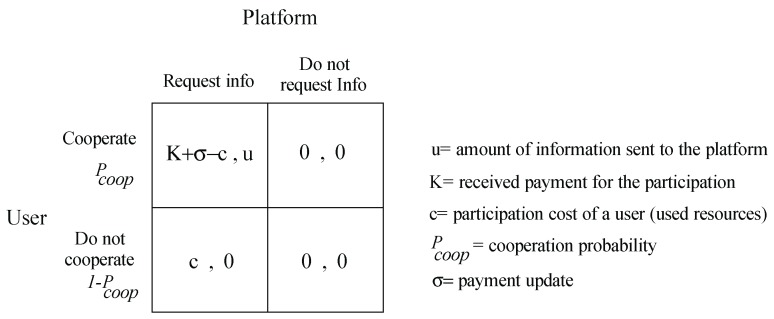
Final representation of our model as normal form game.

Figure 7 shows the final representation of our model as a normal form game.

**Figure 8:**
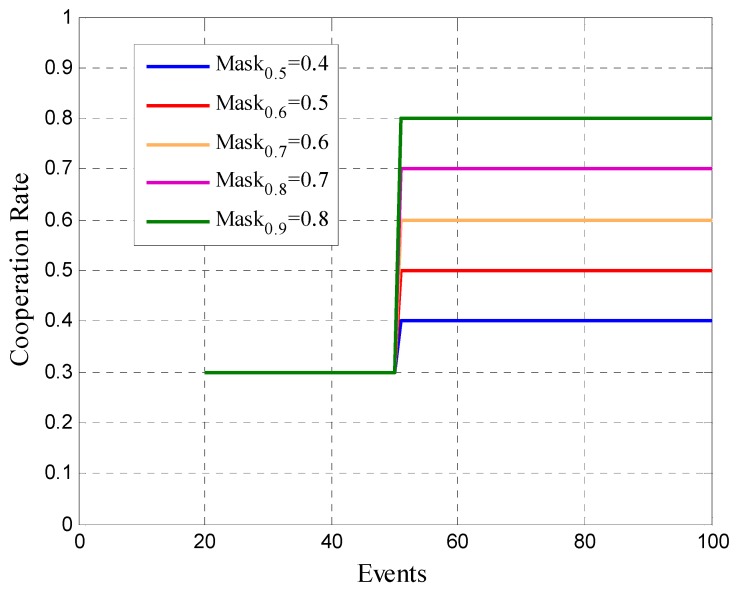
Removal masks.

One goal of the platform is to encourage users to participate constantly in the system. In order to save resources the platform will remove and disregard of its database those who do not have a constant participation. The metric used to decide the removal of users will also be the cooperation rate, but in this case, we will use the moving average of the cooperation rate to consider recent behavior of the variable. Thus, we will have:

(23) $$r_{moving\;}=\frac{1}{n_{i}}\times \overset{n_{i}}{\underset{i=1}{\sum }}r_{i}$$

where $n_{i}$ represents the amount of participation opportunities of user *i* that will be analyzed. $r_{moving\;}$ will be compared at every instant with a removal mask, the same is presented in Figure 8. If the value of $r_{moving\;}$ is below the mask, the platform will remove the user.

We define the removal masks considering the desired cooperation rate of the platform. Analyzing Figure 8, we can see that the masks considers two constraints. The first one is the same for all cases and it can be considered as a soft constraint. The second constraint is more restrictive, has direct relationship with the lower bound of the range of accepted cooperation rate, $lim_{inf}$, and it is equal to $Mask_{r_{desired}}=Lim_{inf_{rdesired}}-0.1$. In the model, we previously set the number of interactions or events that must occur to consider the second constraint of the mask after 50 attempts of participation for all cases. Since, in our simulations this number represents on average half of the interactions between the different users and the platform. A final observation regarding Figure 8 is that in the first interactions between the user and the platform there are no constraints in the different removal masks. Then it is necessary to reach a minimum number of interactions between the platform and the user in order to use the removal criterion.

**Figure 9:**
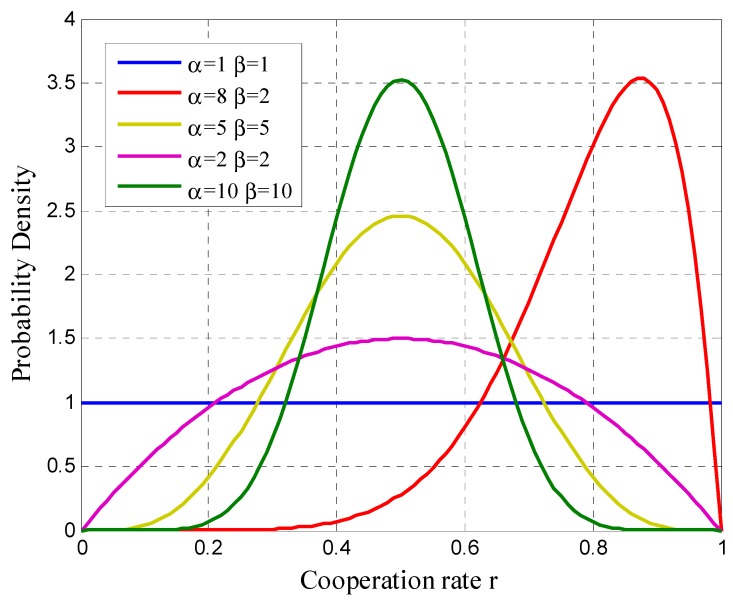
Beta distributions with different α and β.

Note that in the first rounds of participation the platform wants to reach as fast as possible a first estimation of the payment *K* that reflects the participation for each user. The emphasis in the first rounds will be to increase the offered payment *K* each time the cooperation rate *r* decreases in relation to the previous round. If the value of *r* is greater than in the previous round, the platform will repeat the payment on the next participation opportunity. Therefore, we will only perform the update of Equation (13) and the comparison of the cooperation rate with the removal masks after the user has a certain amount of participation attempts. We justify this decision because, as previously expressed, our cooperation rate *r* is considered as the expected value of a Beta probability density function. When the number of interactions (α+β) is very small, the cooperation rate *r* will not be reliable, and consequently the variance between the values is high, which may cause the model to take a wrong decision. Let us analyze a case in which, at the first opportunity of participation the user decides to not participate, his cooperation rate will be 0 but in case he participates the rate would be equal to 1. Trying to predict the behavior of the users in the first instance would be impossible. As the number of observations increase, is possible to have a better perception of their behavior. This is shown in Figure 9 where we can see five Beta distributions with different parameters (α and β).

Note that for cases where α>β, higher values of the parameters will make the probability density narrower and the values that it adopts will be close to the expected value. When α=β=1 the Beta distribution turns into a normal distribution. It can be seen that when α=8 and β=2, the curve shifts to the right indicating a higher cooperation rate.

It is worth pointing out that this incentive mechanism will be limited for applications where the platform threats each user separately. For example, it will not work in applications where users compete for the rewards.

**Figure 10:**
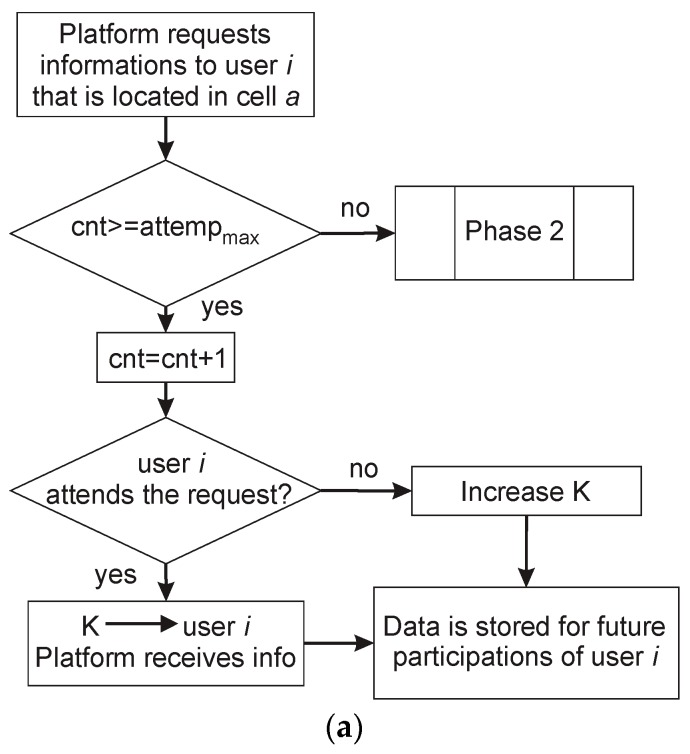

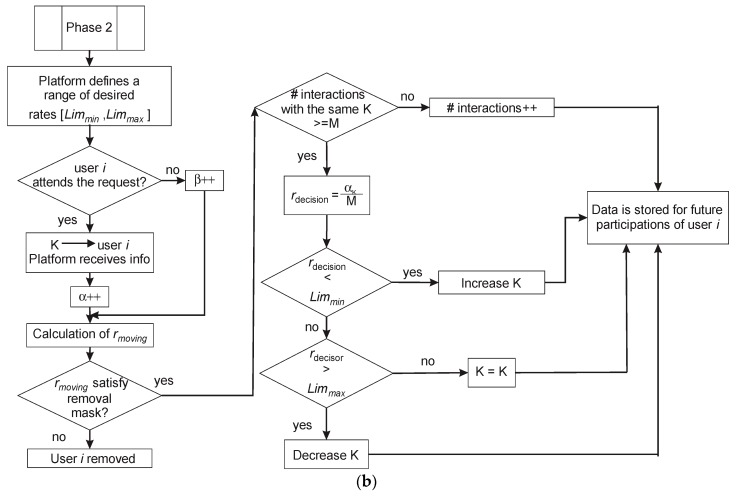
(**a**) Flowchart of the incentive mechanism in its first stage; (**b**) Flowchart of the incentive mechanism in its second stage.

**Figure 11:**
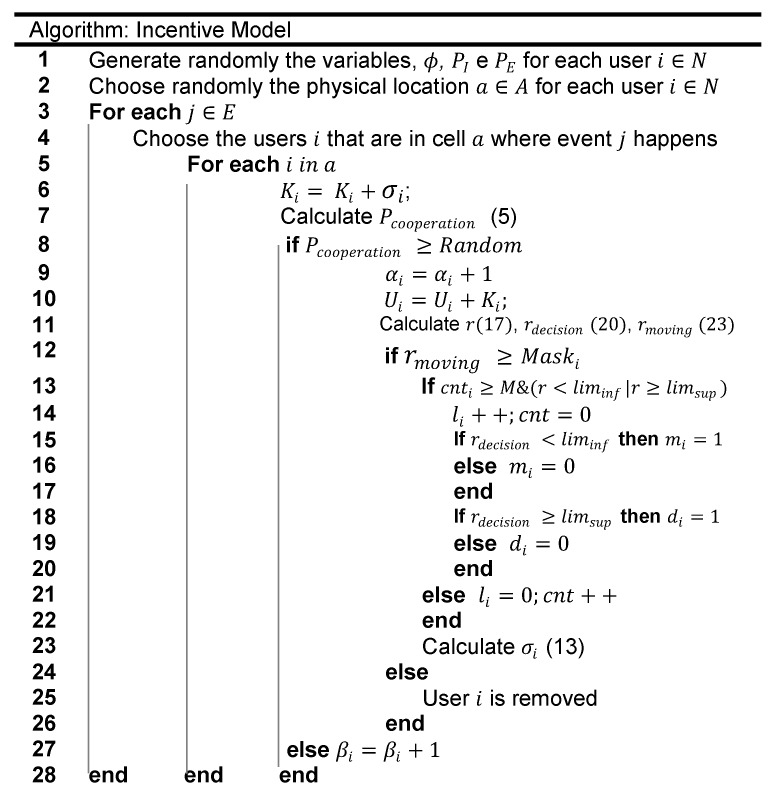
Pseudocode of the incentive mechanism.

We finish this section presenting the flowchart and the pseudocode of the incentive model in Figure 10 and Figure 11 respectively.

## 5. Simulation Results

We conduct simulations in the numerical computing environment MATLAB. Were considered 183 cells or physical locations because is the amount that we need to cover the area of interest with the characteristics shown in Figure 2. This area was chosen given that comprises places of great movement such as tourist areas, financial district, universities, and shopping malls, among others. The initial set of participant users *N* was equal to 1000. The number of platform requirements or events was equal to 13,330 because each user needs a certain amount of interactions with the platform to achieve his stability stage. We previously analyze that at this point we stop seeing modifications in the payments as it will be seen in the results. Events and the locations of users were defined randomly with a uniform distribution. For the calculation of Equation (5) we consider that the values of γ, $\phi ,\;\;P_{I}$ and $P_{E}$ are uniformly distributed random numbers. This will allow us to analyze a scenario where the set of users have different characteristics. The variable that specifies the minimum amount of participation attempts *M* is equal to 10. We perform ten simulations for each fixed combination of parameters and the results presented below are the arithmetic mean of the same.

In Section 5.1 we conducted simulations with four different values of initial payment $Pgto_{0}$, 0.25, 0.5, 0.75 and the maximum payment 1. It is worth pointing that the initial payment $Pgto_{0}$ is the first value of *K*. In other words, we start the negotiation with each user with the value *K*.

In Section 5.2 to analyze and verify if the model manages to reach the platform desired cooperation rate $r_{desired}$, tests were performed with different values of the same. Since, the constants $lim_{inf}$ and $lim_{sup}$ depend directly on the value of $r_{desired}$ they will also take different values for each case of study.

### 5.1. Results for Different Values of Initial Payment with a Fixed Desired Cooperation Rate

**Figure 12:**
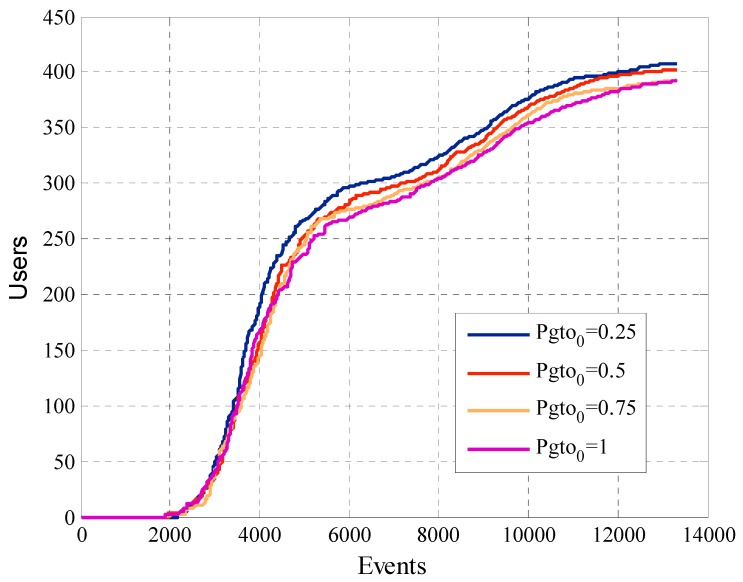
Amount of removed users for configurations with different values of $Pgto_{0}$.

We conducted simulations for the different values of $Pgto_{0}$. All the simulations considered $r_{desired}=0.5$. First, we analyze the number of participant users of the system. In Figure 12 we can see that the number of removed participants does not show a significant or well-defined change for the different configurations of $Pgto_{0}$. The model removes around 40% of the participants in this case. The elimination mask gives the shape of the curve; we can observe that when the number of events increases, the number of removed participants is higher. This is because at this point users had reached the number of participations that allows the model to compare it with the second constraint of the removal mask. The higher the number of events, the greater the likelihood that each user reaches the number of attempts to be compared with the second constraint of the mask.

Once we know the number of participants and removed users, it will be interesting to analyze what characteristics (extrinsic and intrinsic motivations) our set of participant users have. By doing this we will confirm if the mechanism identifies correctly the type of user and makes the right decision for each one; prioritize and consider those who achieve a satisfactory amount of participations and disregard the ones who do not achieve the desired behavior.

**Figure 13:**
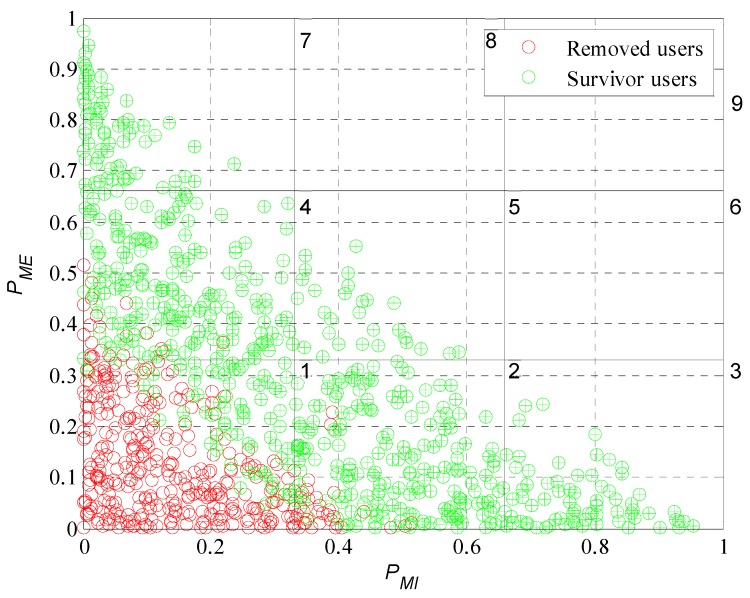
Graphical representation of user motivation levels on a Cartesian plane divided into subgroups.

We will perform a classification considering the probabilities of extrinsic and intrinsic motivation. Both motivations were described in Equations (2) and (3). Each user has an ordered pair, $\left(P_{MI},\;P_{ME}\right)$, that allows us to perform a graphical representation of the levels of motivation of each user in a Cartesian plane divided into subgroups or classes as is illustrated in Figure 13. Analyzing Figure 13 we can see that is not possible to have participant users with their characteristics in the upper diagonal of the plane since as defined in Equation (5) the sum of the extrinsic and intrinsic motivation probabilities cannot be greater than one. Each one of the red and green dots in Figure 13 represent the motivation of a user in Cartesian coordinates. The red ones being those that the model removed of the system during the experiment. The green ones, those that meet the requirements of the platform and are part of the crowd-sensing system. For a better analysis, we enumerate the subclasses; we can see the number of each subclass in the upper right corner of each division. Most of the removed users are in the first subclass because in this subclass users have both of their motivations close to zero. In the second and fourth subclasses are removed users who have one of its characteristics close to zero, the extrinsic in case of the fourth and intrinsic in the second. This analysis has great importance because leads to prove that the incentive model performs correctly the interpretation of the users’ responses to the different incentives. It is able to determine with what type of user he is dealing and if is worthy to continue investing in it. Therefore, the model achieves one of its main objectives; find a set of participants that meet the requirements of the platform.

**Figure 14:**
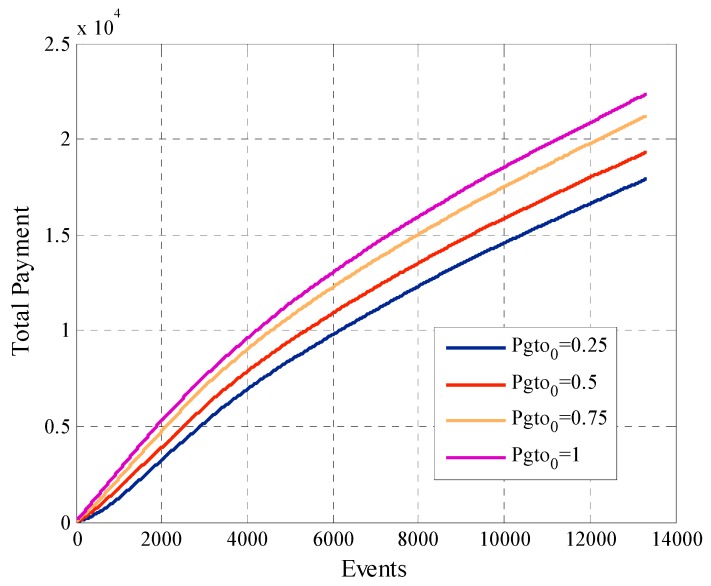
Total payment of the platform for configurations with different values of $Pgto_{0}$.

Figure 14 shows the total payment of the platform. The linear growth of the curves occurs because a considerable number of active users continues to participate when requested. In the first participations, the angular coefficient of the curves is greater since there still are variations in the payments for participation of each user. Once the payments for each user stabilize the slope of the curve decreases and remains constant. The configuration that has $Pgto_{0}=1$ is the one that has the highest total payment although is the one that has the least amount of participant users. This is because when $Pgto_{0}$ is greater, the participation payment converges at a higher value than in the case where $Pgto_{0}$ is lower.

**Figure 15:**
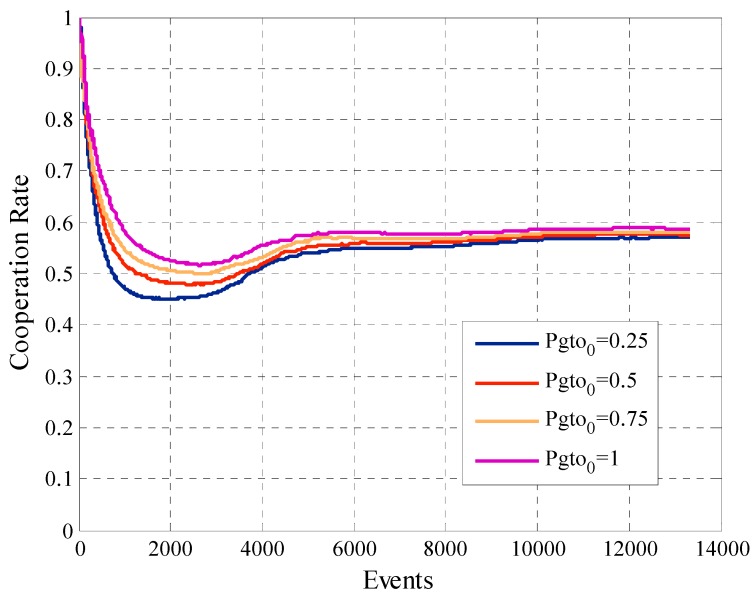
Cooperation rate for configurations with different values of $Pgto_{0}$.

One of the most interesting analysis is related to the cooperation rate. Figure 15 shows the cooperation rate, previously defined in Equation (17), for the different simulations. We can see that all the cases achieve one of the main objectives of the algorithm, ensure that the cooperation rate of the participant user converges within the previously defined range of accepted cooperation rate, which in this case was between the constraints $lim_{inf}=0.5$ and $lim_{sup}=0.6$. With these results, we prove that when the initial payment $Pgto_{0}$ is higher, the cooperation rate is higher too. Although, the difference is minimal between the different cases.

**Figure 16:**
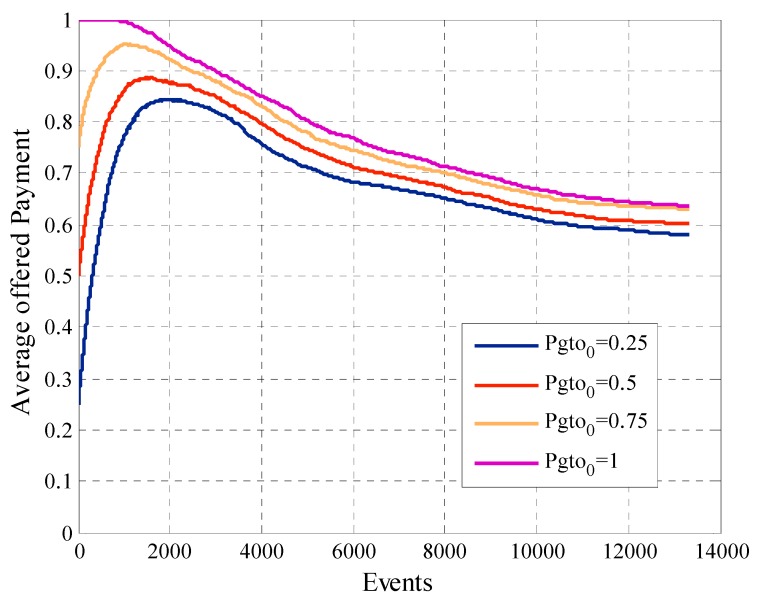
Average offered payment for configurations with different values of $Pgto_{0}$.

Another factor that the system designer must consider is the average offered payment. In Figure 16 we can see that in this case there is a direct relationship between this metric and the initial payment $Pgto_{0}$. Since, when we compare the behavior of two configurations, the one with the highest value of $Pgto_{0}$ will have the average offered payment greater at all times. In the case of the curves with $Pgto_{0}$ equal to 0.25, 0.5 and 0.75 we see how in the first requests of information the average offered payment increases. This is because in the phase one of the mechanism the offered payment increases if the user not participates in the system. When the number of events rises, the platform tries to reduce the payment of the participant users to find the minimum payment that allows the participation for each user. For this type of model, the best option is to start offering a lower payment, since an initial higher payment at the start does not bring greater benefits and raise project costs.

**Figure 17:**
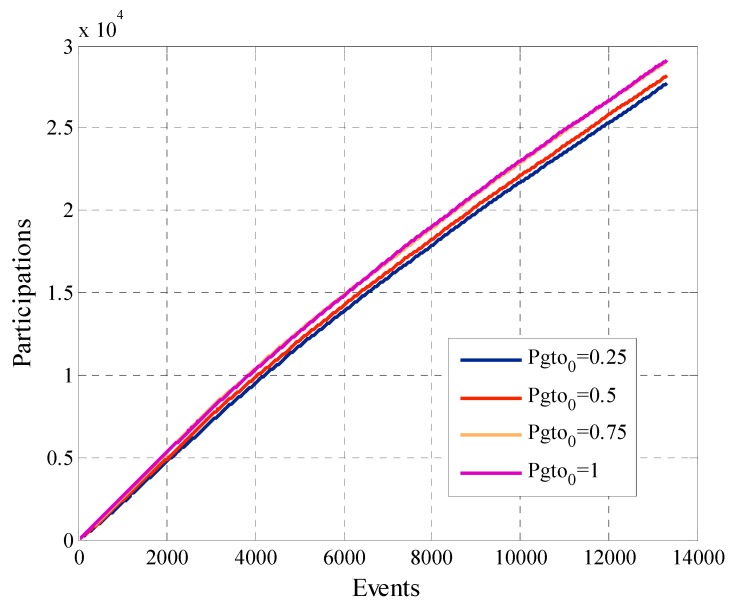
Platform utility for configurations with different values of $Pgto_{0}$.

Figure 17 shows the utility of the platform that was defined in Equation (12). The same represents the total amount of responses that the platform receives from all the participant users. The higher the $Pgto_{0}$, the greater the utility of the platform, but this small gain comes with higher costs as evidenced in Figure 14. We can justify the linear behavior of this metric when we analyze that it represents the cumulative value of the request responses which continue to arrive over time.

### 5.2. Results for Different Values of Desired Cooperation Rate with a Fixed Initial Payment

To have a better understanding of the model, we will analyze the curves for different values of $r_{desired}$. The initial payment $Pgto_{0}$, for all the cases will be equal to 0.5.

**Figure 18:**
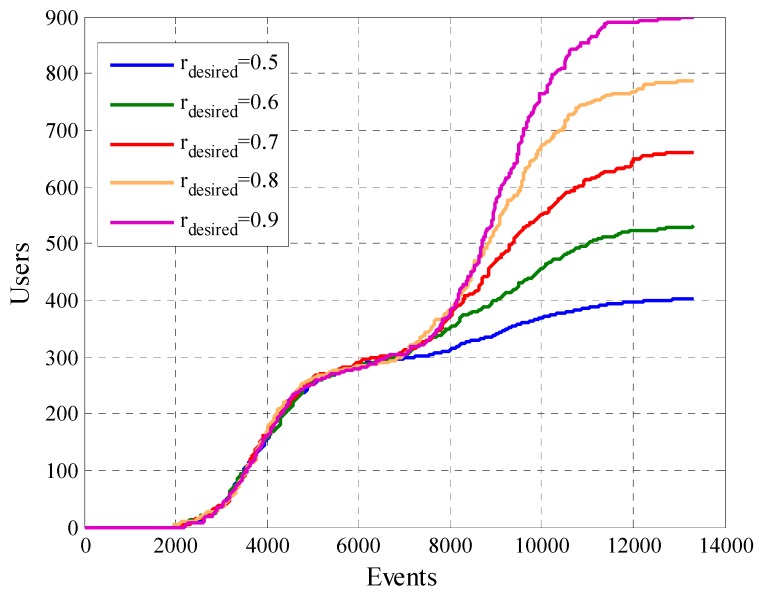
Amount of removed users for configurations with different values of $r_{desired}$.

We will start the analysis of this scenario with the amount of removed users. We see that the number of removed users is higher when $r_{desired}$ is higher. This can be seen in Figure 18 and is mainly because the removed masks are more restrictive when $r_{desired}$ is higher. Few users can participate within these limits.

**Figure 19:**
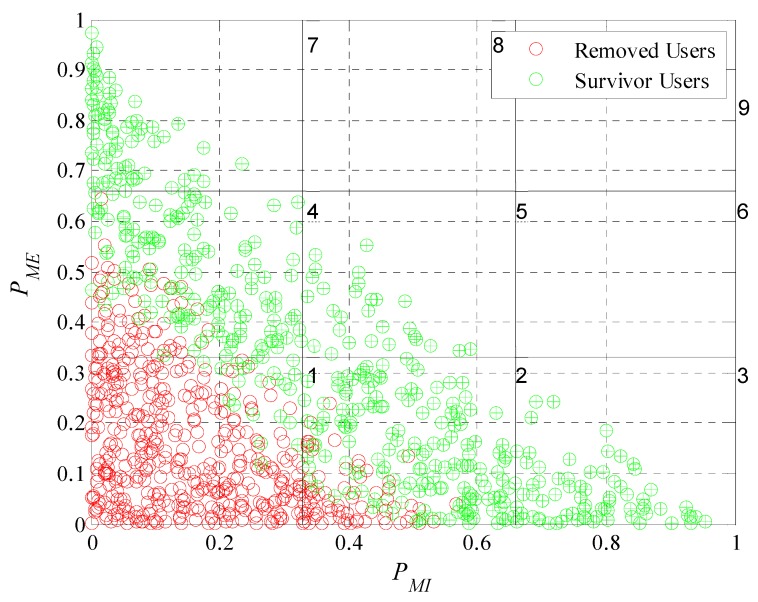
Graphical representation of user motivation levels on a Cartesian plane divided into subgroups when $r_{desired}=0.6$.

**Figure 20:**
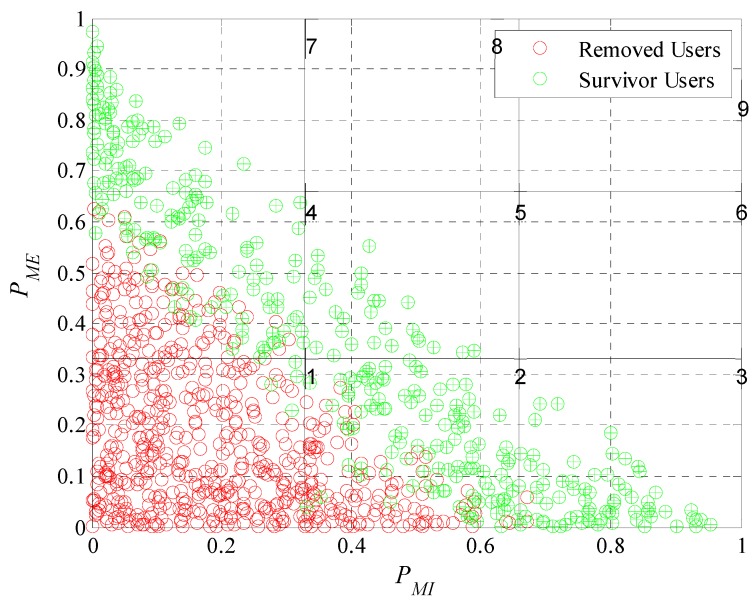
Graphical representation of user motivation levels on a Cartesian plane divided into subgroups when $r_{desired}=0.7$.

**Figure 21:**
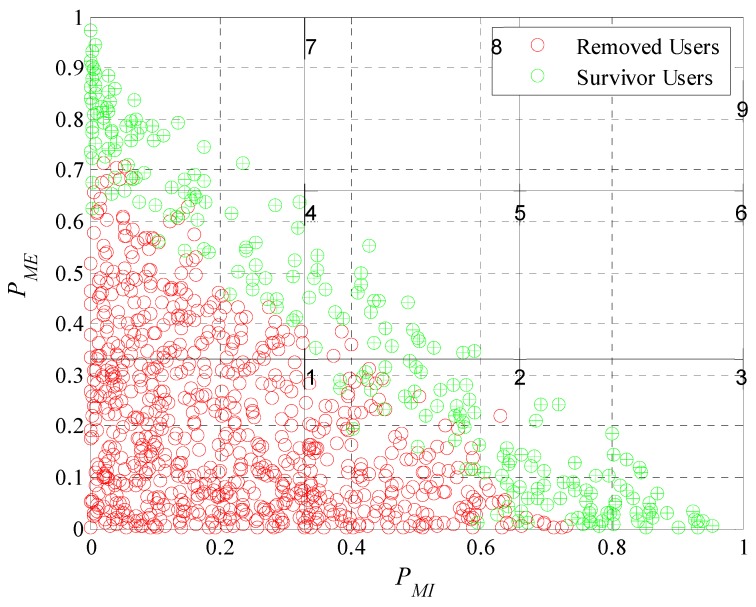
Graphical representation of user motivation levels on a Cartesian plane divided into subgroups when $r_{desired}=0.8$.

**Figure 22:**
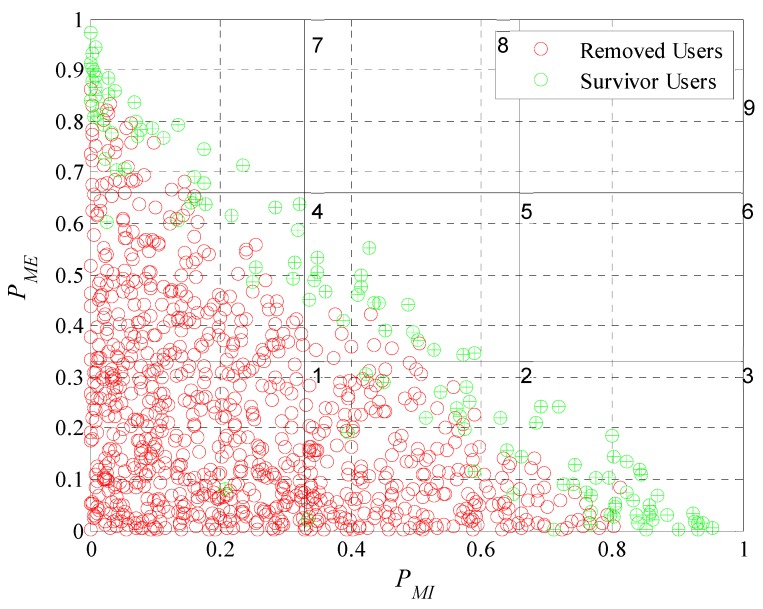
Graphical representation of user motivation levels on a Cartesian plane divided into subgroups when $r_{desired}=0.9$.

We now evaluate the participant and removed users in the subdivision by subclasses as performed in Figure 13. In Figure 19, Figure 20, Figure 21 and Figure 22 we present the analysis for $r_{desired}=0.6,\;0.7,\;0.8$ and 0.9. Noting that we show the case for $r_{desired}=0.5$ in Figure 13. With this set of Figures, we aim to reinforce the conclusion obtained through Figure 18. The higher the value of $r_{desired}$ the greater the number of removed users. We can observe that users that the model considers in a scenario of lower $r_{desired}$ will be removed when the scenario has higher requirements. The characteristics of the survivor users (green dots) moves further away from the origin when $r_{desired}$ is higher, converging on the diagonal of the plane. As in Figure 13, we can verify for the different values of $r_{desired}$ that the incentive model determines and chooses correctly the users that best adapt to the initial requirements of the platform. We also prove that in a population with different types of users is complex to see many users who meet strict requirements.

In these graphical representations, we can also observe the presence of some specific errors in the identification of the type of user, as in Figure 22, where there is a participant user in the lower left quadrant that the platform should have removed. We can define that an error occurs when: (i) A user is considered an active participant having his probability of cooperation lower than the restrictive criterion of the removal mask or when (ii) a user is removed having his probability of cooperation greater than the restrictive criterion of the removal mask. Remembering that we define the probability of cooperation in Equation (5) and is equal to the sum of $P_{MI}$ and $P_{ME}$. Table 2 shows the number of errors for each case. In the first column we see the different values use for $r_{desired}$. In the second column, we tabulated the removal errors (users erroneously removed). While in the third column, the participation errors (users erroneously active) can be found. In the last column of Table 2 we can see the percentage of errors occurred in relation to the total number of users. With this data it was possible to show that the incentive model can correctly determine the type of user over 92% of the times for the case where $r_{desired}=0.5$. In the case of $r_{desired}=0.9$ the model determines correctly the type of user more than 95% of the times. Proving that the number of errors is small when compared to the total number of decisions taken. It is also proven that when $r_{desired}$ is higher the amount of errors will be lower.

**Figure 23:**
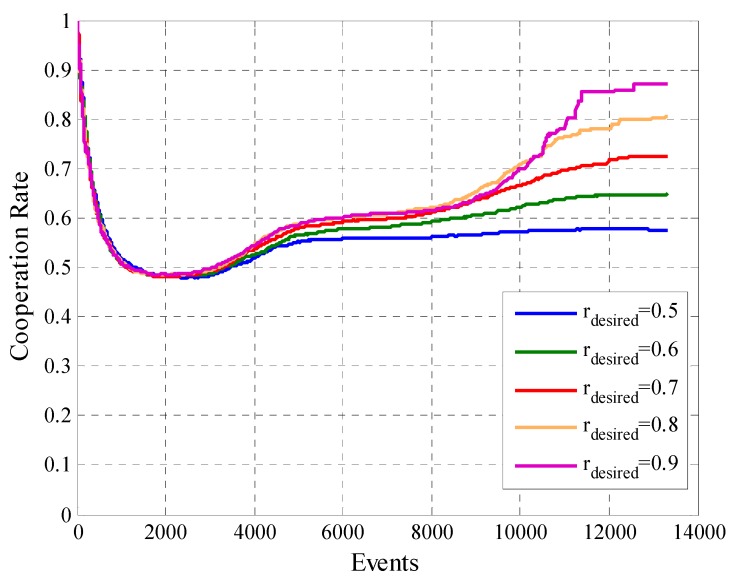
Cooperation rate for configurations with different values of $r_{desired}$.

**Figure 24:**
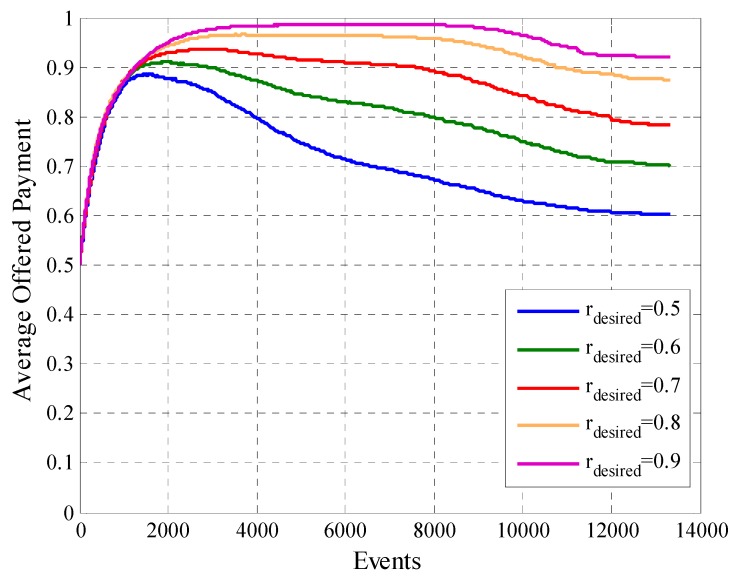
Average offered Payment for configurations with different values of $r_{desired}$.

In Figure 23 we observe that the model fulfills its previously defined objectives for each $r_{desired}$ since for each case the cooperation rate reaches the range defined by its respective $lim_{inf}$ and $lim_{sup}$. The average offered payment to users for reaching the desired cooperation rate for each case is in Figure 24. In the same, we can observe that when $r_{desired}$ is higher the average offered payment is greater. This happens throughout the experiment for all the cases. We also observe that the variable $r_{desired}$ determines how much it is possible to decrease the payment of the users. In the case that $r_{desired}$ has higher values, the average offered payment has a smaller decrease than in the case that $r_{desired}$ has lower values.

**Figure 25:**
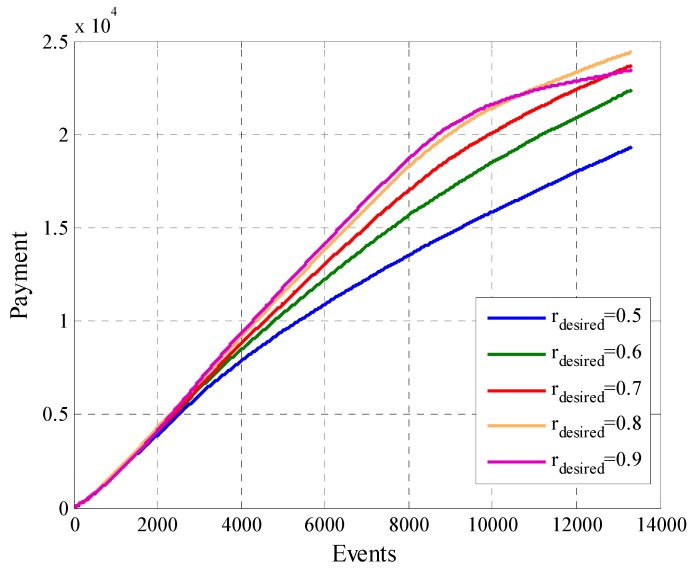
Total Payment for configurations with different values of $r_{desired}$.

**Figure 26:**
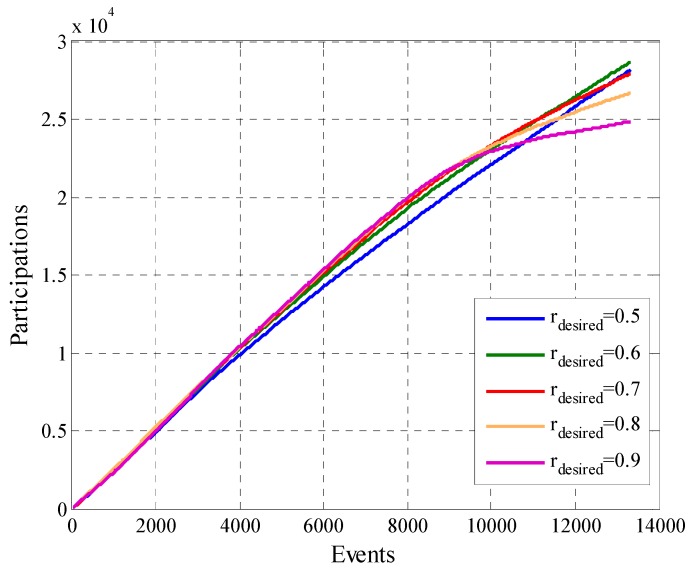
Platform utility for configurations with different values of $r_{desired}$.

Finally, we will analyze the total payment of the system in Figure 25 and the utility of the platform in Figure 26. In Figure 25 we can see that configurations with a higher value of $r_{desired}$ result in a higher cost for most time of the experiment than those with a lower $r_{desired}$. This behavior changes in the final part of the experiment when all the participant users reached enough opportunities to compare their cooperation rate with the second removal mask criterion. Scenarios where the value of $r_{desired}$ is high have a bigger number of removed users (Figure 17), which reflects in a considerable drop in the total system payment as can be seen in Figure 25 in the cases where $r_{desired}=0.8$ and $r_{desired}=0.9$. A similar behavior is show in the case of the total utility of the platform in Figure 26, in which likewise the curves of cases where $r_{desired}=0.8$ and $r_{desired}=0.9$ are affected by the decrease of the number of participant users. The scenario that defines $r_{desired}=0.6$ achieves the better utility, this is mainly because to the balance between the characteristics of participant users and the amount of them.

## 6. Summary and Conclusions

In this paper, we present a framework for MCS that includes a model to represent the behavior of users and a novel incentive mechanism. The model for the characterization of the behavior of users addresses the availability of users’ resources and the non-homogeneity of their responses. The incentive mechanism proposed assigns different values of incentives considering the unique characteristics of each user.

Incentive solutions presented in other studies consider the non-participation of a user as a strategic behavior and that a user needs the information of other users to decide its participation in the system. In this case, the proposed incentive mechanism achieves their objectives considering only local (individual) information. The simulations results validated the uncertainty of participation of the users and that they react in different ways to the incentives offered. They also prove that the incentive mechanism estimates satisfactorily the type of users and the incentive it will offer to each user just with one information, the responses of users to the different incentives values. It is worth noting that one of the outstanding feature of the incentive mechanism is that it manages to converge the cooperation rate of the system to the desired by the platform. The mechanism performs a correct selection of users since at the end of each study case the participant users are those that reach the platform requirements. Finally, we prove that starting the interaction with the users through a fixed maximum payment will bring unnecessary expenses. Using variable payments allows savings in the total cost of the system.

Modeling important characteristics of human behavior and MCS allows researchers to perform tests in different scenarios to have a better analysis of this new sensing paradigm before testing these features in a real system.

For future work, it will be interesting implement the proposed solution in a real deployment case. Another important issue will be to modify the incentive mechanism to limit the value of the incentives considering that the platform has a budget for the total payment of the system. We plan to analyze how the incentive model performs considering other particular characteristics of these type of approaches, such as the human mobility. An additional line of research is to find methods to make a fair comparison between our model and other solutions already proposed. The framework developed here can serve as a basis for further implementations and analyzes.

## Figures and Tables

**Table 1:** Notation definitions.

Notations	Definition	Notations	Definition
$P_{I}{}_{i}$	Probability of cooperation since the motivation is intrinsic	$P_{E}{}_{i}$	Probability of cooperation since the motivation is extrinsic
$P_{MI}{}_{i}$	Intrinsic motivation probability	$P_{ME}{}_{i}$	Extrinsic motivation probability
$\gamma_{i}$	Weight of intrinsic motivation	$\phi_{i}$	Weight of extrinsic motivation
$K_{i}$	Incentive	c	Participation cost
$P_{cooperation}{}_{i}$	Cooperation probability	$K_{Total}$	Total cost of the system
$U_{i}$	Total utility of the user	$U_{p}$	Total quantity of responses
σ	Offered payment update	$sta_{i}$	Stability criterion
inc	Maximum value of increment for *K*	dim	Maximum value of diminution for *K*
$r_{i}$	Cooperation rate	cnt	Amount of participation attempts
$l_{i}$	Minimum number of interactions between user and platform	M	Minimum amount of participation attempts
$m_{i}$	Flag for increasing the payment	$d_{i}$	Flag for decreasing the payment
$lim_{inf}{}_{i}$	Lower limit of cooperation rate	$\;lim_{sup}{}_{i}$	Upper limit of cooperation rate
$r_{desired}$	Percentage of participation that the platform wants to achieve	$r_{decision}$	Behavior of the user to the fixed payment
$r_{moving\;}$	Percentage of recent participation		

**Table 2:** Analysis of the number of errors in the identifications of the type of user.

$r_{desired}$	Amount of Elimination Errors	Amount of Participation Errors	Total Amount of Errors	Percentage of Errors
0.5	56	23	79	7.9%
0.6	48	24	72	7.2%
0.7	45	27	72	7.2%
0.8	35	23	58	5.8%
0.9	27	22	49	4.9%
